# Temporary at-Rest Myocardial Perfusion Defect: A Possible Case of Takotsubo Syndrome

**Published:** 2018-01

**Authors:** Saeed Farzanefar, Sanaz Katal, Mehrshad Abbasi

**Affiliations:** 1 *Department of Nuclear Medicine, Vali-Asr Hospital, Tehran University of Medical Sciences, Tehran, Iran. 1419731351. Tel: + 98 21 61192405. Fax: +98 21 66575103. E-mail: farzanefar@tums.ac.ir.*; 2 *Research Institute for Nuclear Medicine, Shariati Hospital, Tehran University of Medical Sciences, Tehran, Iran. 1411713135. Tel: + 98 21 88953001. Fax: + 98 21* *88633039. E-mail: sanaz.katal@gmail.com.*; 3 *Associate Professor of Nuclear Medicine, Department of Nuclear Medicine, Vali-Asr Hospital, Tehran University of Medical Sciences, Tehran, Iran. 1419731351. Tel: + 98 21 61192405. Fax: +98 21 66575103. E-mail: * *meabbasi@tums.ac.ir* *.*

Broken-heart syndrome is an uncommon nonischemic cardiomyopathy with temporary myocardial infarction-like symptoms and signs with normal angiography^1^^, ^^2^ but presumably coronary microvasculature abnormalities.^2^^, ^^3^ The condition is a transient dysfunction and stunning of the mainly apical segments of the myocardium of the left ventricle.^1^^, ^^4^^, ^^5^ A major stressor is detected in the majority of the patients,^6^ who are predominantly postmenopausal females.^7^ The functional abnormalities tend to completely improve within days to months.^1^^, ^^8^ There are metabolic changes in the myocardium confirmed by ^18^F-fluorodeoxyglucose positron emission tomography (^18^F-FDG PET), indicating the cellular dysmetabolism of glucose without significant ischemia.^9^^-^^11^ The underlying cause could be adrenergic denervation assessed by iodine-123-metaiodobenzylguanidine single-photon emission computed tomography (^123^I-mIBG SPECT).^11^^, ^^12^ The ischemic component of the condition has been suggested by myocardial perfusion imaging (MPI) in a few cases.^13^^, ^^14^

**Figure 1:**
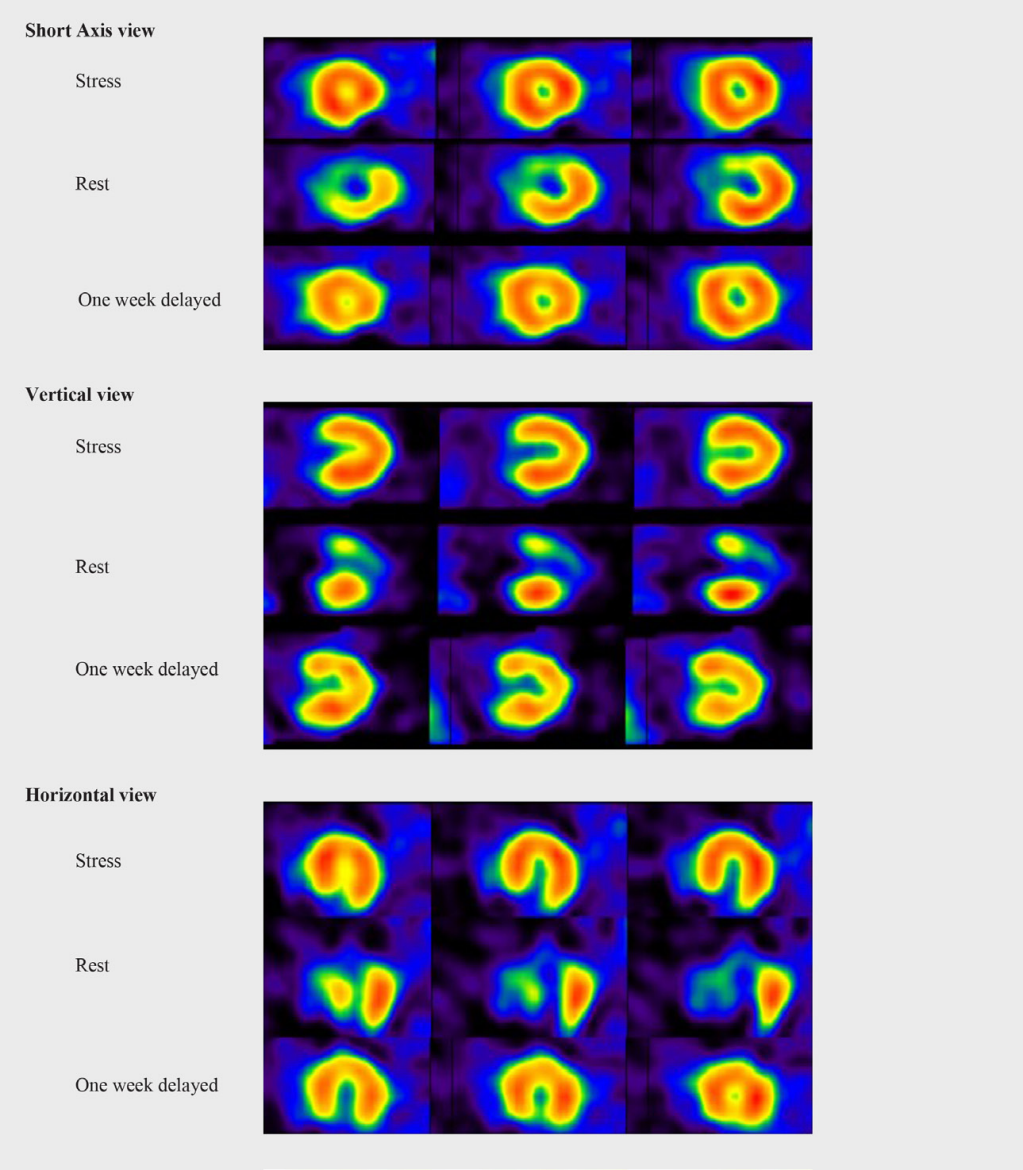
Myocardial perfusion single-photon emission computed tomography of a 52-year-old patient with temporary at-rest perfusion defects (Takotsubo syndrome); normal stress (dipyridamole), perfusion defect at the anterior, anteroseptal, and apical myocardial walls at rest (^99m^Tc-MIBI) and normal 1 week’s delayed repeat rest images (Tl-201).

We performed a 2-day stress and rest MPI with technetium sestamibi (^99m^Tc-MIBI) for a diabetic woman aged 52 years. She was consulted for cardiac risk assessment. The stress was performed pharmacologically via a slow infusion (4 min) of 0.56 mg/kg of dipyridamole for the first-day study. There was no remarkable abnormality at stress MPI, with normal myocardial motion and contractility and a normal left ventricular ejection fraction. On the next day, rest MPI presented significant perfusion defects at the anterior, anteroseptal, and apical myocardial walls (Figure 1). The patient denied any cardiac-related symptoms, and her clinical examination and electrocardiogram were normal. She reported emotional stress; nevertheless, her stressor was not significant. We followed her up for 7 days, after which her rest MPI was repeated by thallium-201. The perfusion abnormality noticed in prior rest MPI had disappeared in these images (Figure 1). 

The patient had normal stress myocardial perfusion, but the rest images on the next day presented perfusion defects reversible at 1 week’s delayed repeated rest images. This is one of the very few reports of at-rest temporary MPI abnormalities, which could be classified as broken-heart syndrome or Takotsubo syndrome. The underlying pathology is assumed to be in the micro-coronary vasculature, which is not reflected in the angiogram of coronary arteries.^4^^, ^^5^ MPI shows the perfusion of the myocardium at cellular level and may reflect macro- or micro-coronary or functional abnormalities.^13^^-^^15^ Broken-heart syndrome is by definition a nonischemic cardiomyopathy.^16^ Our MPIs indicated a major temporary perfusion abnormality in this case. To the best of our knowledge, at-rest perfusion abnormalities could be due to myocardial infarction, hibernation, or artifacts including attenuation, non-uniformity, and center-of-rotation artifacts. We excluded the mentioned artifacts, which cause false perfusion abnormalities, by reviewing the raw cinematic images. Considering that our patient’s perfusion abnormality improved after 1 week, the only remaining possible diagnosis was Takotsubo syndrome. She had no acute coronary symptoms; hence, we did not determine myocardial enzymes and did not perform angiography. Diagnosis of Takotsubo syndrome requires that its criteria be fulfilled; we failed to provide sufficient evidence to confirm the diagnosis. Whether or not the present patient can be considered a case of Takotsubo syndrome, this paper is a unique report of MPI studies with reversible at-rest perfusion abnormalities with normal prior stress myocardial perfusion.
